# Nitrative stress activates JNK and decreases retrograde transport of brain‐derived neurotrophic factor in basal forebrain cholinergic neurons

**DOI:** 10.1002/alz70855_104239

**Published:** 2025-12-23

**Authors:** Jessica Fisher, Sama Jaberi, Erika Kropf, Margaret Fahnestock

**Affiliations:** ^1^ McMaster University, Hamilton, ON, Canada

## Abstract

**Background:**

Basal forebrain cholinergic neurons (BFCNs) lose synapses and degenerate with age and in Alzheimer's disease (AD), contributing to cognitive decline. BFCNs rely on retrograde axonal transport of brain‐derived neurotrophic factor (BDNF), a neurotrophin essential for synaptic plasticity, to maintain learning and memory. BDNF transport is decreased in aging, but the mechanisms are unclear. Elevated levels of nitrative stress occur in the aging brain and may promote BFCN neurodegeneration by interfering with BDNF retrograde transport. This study evaluates whether nitrative stress‐induced activation of c‐Jun *N*‐terminal kinase (JNK) contributes to BDNF transport deficits.

**Method:**

Primary rat BFCNs cultured for 9 days in microfluidic chambers (creating fluidic isolation between axon terminals and cell bodies) were treated with either 1) a peroxynitrite donor (SIN‐1) or 2) a combination of a JNK inhibitor (CC401) and SIN‐1. JNK activation was measured by immunocytochemistry and BDNF axonal transport was assessed by adding Quantum dot‐labelled BDNF to axon terminals followed by live cell fluorescence microscopy at the proximal axons (near cell bodies).

**Result:**

SIN‐1 alone increased JNK activation and decreased BDNF transport, while inhibition of JNK with CC401 rescued SIN‐1‐associated transport deficits.

**Conclusion:**

This suggests that JNK activation by peroxynitrite may be a mechanism by which nitrative stress reduces BDNF transport in BFCNs. This study reveals that age‐related nitrative stress may contribute to BDNF transport deficits, leading to BFCN degeneration and cognitive decline in AD.

